# Annual Meeting of the Chicago Dental Society

**Published:** 1892-05

**Authors:** Geo. J. Dennis

**Affiliations:** Cor. Sec'y.


### ANNUAL MEETING OF THE CHICAGO DENTAL SOCIETY.

At the annual meeting of the Chicago Dental Society, held Tuesday evening, April 5th, 1892, the following officers were elected for the ensuing year :

J. W. Wassall, president; Thos. L. Gilner, first vice-president; E. A. Royce, second vice-president; L. L. Davis, recording secretary; Geo. J. Dennis, corresponding secretary; E. D. Swain, treasurer; J. H. Smyzer, librarian; G. H. Cushing, E. Noyes, J. G. Reid, board of directors; A. H. Peck, B. D. Wikoff, D. M. Gallie, board of censors.

GEO. J. DENNIS, *Cor. Sec'y.*

